# Influenza and COVID-19 Vaccination Coverage Among Health Care Personnel — United States, 2021–22

**DOI:** 10.15585/mmwr.mm7142a2

**Published:** 2022-10-21

**Authors:** Hilda Razzaghi, Anup Srivastav, Marie A. de Perio, A. Scott Laney, Carla L. Black

**Affiliations:** ^1^Immunization Services Division, National Center for Immunization and Respiratory Diseases, CDC; ^2^Leidos, Inc., Atlanta, Georgia; ^3^Office of the Director, National Institute for Occupational Safety and Health, CDC; ^4^Division of Respiratory Health, National Institute for Occupational Safety and Health, CDC.

The Advisory Committee on Immunization Practices (ACIP) and CDC recommend that all health care personnel (HCP) receive annual influenza vaccination to reduce influenza-related morbidity and mortality among these personnel and their patients (*1*). ACIP also recommends that all persons aged ≥6 months, including HCP, be vaccinated with COVID-19 vaccines and remain up to date (*2*,*3*). During March 29–April 19, 2022, CDC conducted an opt-in Internet panel survey of 3,618 U.S. HCP to estimate influenza vaccination coverage during the 2021–22 influenza season as well as receipt of the primary COVID-19 vaccination series and a booster dose. Influenza vaccination coverage was 79.9% during the 2021–22 season, and 87.3% of HCP reported having completed the primary COVID-19 vaccination series; among these HCP, 67.1% reported receiving a COVID-19 booster dose. Among HCP, influenza, COVID-19 primary series, and COVID-19 booster dose vaccination coverage were lowest among assistants and aides, those working in long-term care (LTC) or home health care settings, and those whose employer neither required nor recommended the vaccines. Overall, employer requirements for influenza and COVID-19 primary series vaccines were reported by 43.9% and 59.9% of HCP, respectively; among HCP who completed the primary series of COVID-19 vaccines, 23.5% reported employer requirements for COVID-19 booster vaccines. Vaccination coverage for all three vaccine measures was higher among HCP who reported employer vaccination requirements and ranged from 95.8% to 97.3% for influenza, 90.2% to 95.1% for COVID-19 primary series, and 76.4% to 87.8% for COVID-19 booster vaccinations among HCP who completed the primary series of COVID-19 vaccines, by work setting. Implementing workplace strategies demonstrated to improve vaccination coverage among HCP, including vaccination requirements or active promotion of vaccination, can increase influenza and COVID-19 vaccination coverage among HCP and reduce influenza and COVID-19–related morbidity and mortality among HCP and their patients (*4*).

An Internet panel survey of HCP was conducted during March 29–April 19, 2022, to provide estimates of influenza and COVID-19 vaccination coverage among HCP during the 2021–22 influenza season. Similar surveys have been conducted annually since the 2010–11 influenza season, and previously published results from the 2020–21 season are available (*5*). Respondents were recruited from two preexisting national opt-in Internet sources: Medscape,* a medical website managed by WebMD Health Professional Network, and general population Internet panels operated by Dynata.^†^ Responses were weighted to the distribution of the U.S. population of HCP^§^ by occupation,^¶^ age, sex, race and ethnicity, work setting, and U.S. Census Bureau region. A poststratification weight for each survey respondent was calculated by fitting a generalized exponential model and estimating the model parameters using calibration equations (*6*). Among 3,830 eligible participants, a total of 3,679 completed the survey (completion rate = 96.1%**). Sixty-one participants were excluded because they did not report their occupational setting or indicated a setting other than those listed, and the verbatim description did not qualify as a health care setting, leaving 3,618 respondents in the analytic sample. This activity was reviewed by CDC and was conducted consistent with applicable federal law and CDC policy.^††^

Estimated (weighted) proportions and corresponding 95% CIs for three vaccination measures (influenza vaccination coverage for the 2021–22 season, COVID-19 vaccination coverage [i.e., receipt of ≥1 dose and completion of primary series^§§^], and COVID-19 booster vaccination^¶¶^) were estimated for each work setting, occupation, and demographic characteristic. LTC settings include nursing homes, assisted living facilities, other long-term care facilities, home health agencies, and home health care. Employer requirements for all three vaccination measures were assessed through three separate questions.*** The Korn-Graubard method was used to calculate CIs for proportions, assuming that the weighted estimates were approximately unbiased.^†††^ CDC’s National Center for Health Statistics reliability criteria for proportions were applied to the estimates in the descriptive analyses of HCP characteristics (*7*). T-tests were used to assess differences among subgroups; p<0.05 was considered statistically significant. SAS (version 9.4; SAS Institute) and SAS-callable SUDAAN (version 11.0.1; RTI International) were used to conduct all analyses.

Overall, 79.9% of HCP reported having received an influenza vaccination during the 2021–22 season, not significantly different from the 75.9% reported during the 2020–21 season (Table 1). During the 2021–22 season, higher influenza vaccination coverage was reported among HCP with either a master’s, professional, or doctoral degree (92.3%) and an associate or bachelor’s degree (80.2%) than among those with some college education or less (66.7%). Influenza vaccination coverage was lower among nurse practitioners and physician assistants (92.4%), nurses (87.8%), other clinical personnel (87.8%), nonclinical HCP (75.7%), and assistants and aides (68.8%) compared with coverage among physicians (96.8%). Influenza vaccination coverage during 2021–22 was highest among HCP working in hospitals (92.0%) and lowest among HCP working in LTC settings (66.4%). Coverage was higher among HCP who reported an employer requirement for influenza vaccination (96.8%) than among those who reported an employer recommendation (76.5%) or no recommendation or requirement for vaccination (48.1%). Compared with the 2020–21 influenza season, increases in influenza vaccination coverage were observed among HCP aged 30–45 years (7.5 percentage points), those with more than a college degree (10.9 percentage points), physicians (5.5 percentage points), and pharmacists (4.3 percentage points).

**TABLE 1:** Receipt of influenza vaccination during 2020–21 and 2021–22 influenza seasons among health care personnel, by selected characteristics — Internet panel surveys,* United States, April 2021 and April 2022

Characteristic	2020–21 Influenza season		2021–22 Influenza season		Percentage point change in weighted % vaccinated, 2020–21 to 2021–22 (95% CI)^†^
	No. (weighted %)	Weighted % vaccinated (95% CI)^†^	No. (weighted %)	Weighted % vaccinated (95% CI)^†^	
**Total**	**2,391**	**75.9 (71.3 to 80.1)**	**3,618**	**79.9 (76.6 to 82.9)**	**4.0 (−1.4 to 9.4)**
**Age group, yrs**					
18–30 (Ref)	263 (17.5)	65.0 (48.1 to 79.5)^§^	343 (17.3)	71.4 (55.7 to 84.0)	6.4 (−14.7 to 27.5)
30–45	1,007 (38.9)	76.3 (69.8 to 82.0)	1,616 (39.7)	83.8 (80.8 to 86.5)	7.5 (0.8 to 14.2)^¶^
45–60	774 (29.0)	79.2 (72.0 to 85.3)	1,112 (29.1)	77.7 (73.6 to 81.5)	−1.5 (−9.2 to 6.2)
≥60	346 (14.6)	81.3 (71.2 to 89.0)	547 (13.9)	83.7 (77.5 to 88.8)	2.4 (−8.1 to 12.9)
Race and ethnicity**					
White, non-Hispanic (Ref)	1,419 (61.4)	79.9 (75.1 to 84.1)	2,329 (60.7)	80.9 (76.6 to 84.7)	1.0 (−5.1 to 7.1)
Black, non-Hispanic	316 (17.0)	67.4 (52.9 to 79.9)	319 (16.5)	77.3 (70.3 to 83.3)	9.9 (−5.1 to 24.9)
Hispanic or Latino	399 (14.1)	68.0 (48.5 to 83.8)^§^	485 (14.3)	78.5 (65.5 to 88.3)	10.5 (−10.5 to 31.5)
Other, non-Hispanic	253 (7.5)	77.1 (62.7 to 87.9)	471 (8.5)	80.4 (71.9 to 87.2)	3.3 (−11.4 to 18.0)
**Education**					
Some college education or less (Ref)	541 (29.1)	66.7 (58.2 to 74.6)	526 (27.3)	66.7 (59.9 to 73.1)	0.0 (−10.5 to 10.5)
Associate or bachelor’s degree	767 (45.2)	78.7 (70.7 to 85.3)**^††^**	1,038 (45.0)	80.2 (74.3 to 85.2)**^††^**	1.5 (−7.6 to 10.6)
Master’s, professional, or doctoral degree	1,082 (25.7)	81.4 (74.4 to 87.1)**^††^**	2,053 (27.7)	92.3 (89.7 to 94.5)**^††^**	10.9 (4.1 to 17.7)^¶^
**Occupation** ^§§^					
Physician (Ref)	283 (3.4)	91.3 (85.2 to 95.5)	591 (3.6)	96.8 (94.9 to 98.1)	5.5 (0.1 to 10.9)^¶^
Nurse practitioner/Physician assistant	147 (1.4)	88.9 (56.0 to 99.5)^§^	333 (1.7)	92.4 (88.7 to 95.1)**^††^**	3.5 (−18.5 to 25.5)
Nurse	179 (18.4)	90.3 (82.2 to 95.5)	362 (18.7)	87.8 (82.7 to 91.8)**^††^**	−2.5 (−10.6 to 5.6)
Pharmacist	309 (1.3)	90.3 (86.4 to 93.4)	509 (1.5)	94.6 (92.2 to 96.4)	4.3 (0.2 to 8.4)^¶^
Other clinical personnel^¶¶^	561 (18.8)	83.0 (75.5 to 89.0)**^††^**	916 (18.8)	87.8 (85.2 to 90.1)**^††^**	4.8 (−2.4 to 12.0)
Assistant/Aide	577 (24.2)	64.8 (60.4 to 68.9)**^††^**	540 (24.8)	68.8 (64.4 to 73.0)**^††^**	4.0 (−2.0 to 10.0)
Nonclinical personnel***	306 (32.5)	69.0 (55.8 to 80.2)**^††^**	333 (30.9)	75.7 (65.9 to 83.9)**^††^**	6.7 (−8.5 to 21.9)
**Work setting** ^†††^					
Hospital	914 (38.8)	91.6 (87.8 to 94.5)**^††^**	1,488 (40.3)	92.0 (89.6 to 94.1)**^††^**	0.4 (−3.6 to 4.4)
Ambulatory care	734 (22.8)	77.3 (63.9 to 87.6)	1,335 (31.2)	81.2 (77.2 to 84.7)	3.9 (−8.5 to 16.3)
Long-term care facility/Home health care^§§§^	576 (41.6)	66.0 (57.6 to 73.6)**^††^**	646 (29.3)	66.4 (57.5 to 74.4)**^††^**	0.4 (−11.0 to 12.0)
Other clinical setting^¶¶¶^	629 (10.9)	66.8 (54.6 to 77.5)	754 (9.5)	79.4 (72.4 to 85.3)	12.6 (−0.5 to 25.7)
Location of primary workplace****					
Rural (Ref)	308 (12.2)	71.6 (60.1 to 81.4)	496 (14.8)	76.5 (70.7 to 81.6)	4.9 (−7.1 to 16.9)
Nonrural	2,080 (87.8)	76.5 (71.3 to 81.2)	3,117 (85.2)	80.5 (76.7 to 83.9)	4.0 (−2.1 to 10.1)
**U.S. Census Bureau region** ^††††^					
Northeast (Ref)	456 (19.8)	83.6 (76.5 to 89.2)	791 (19.9)	84.0 (79.0 to 88.1)	0.4 (−7.4 to 8.2)
Midwest	399 (23.3)	73.9 (63.3 to 82.9)	816 (23.1)	82.8 (78.5 to 86.4)	8.9 (−1.7 to 19.5)
South	1,024 (36.1)	75.5 (67.5 to 82.3)	1,251 (35.8)	77.7 (70.8 to 83.7)	2.2 (−7.6 to 12.0)
West	507 (20.8)	71.5 (57.5 to 83.1)	760 (21.1)	76.5 (67.7 to 84.0)	5.0 (−10.2 to 20.2)
**Employer influenza vaccination requirement**					
Required (Ref)	843 (34.2)	95.9 (92.6 to 98.0)	1,714 (43.9)	96.8 (95.3 to 98.0)	0.9 (−2.1 to 3.9)
Recommended	1,024 (42.4)	76.2 (69.9 to 81.8)**^††^**	1,293 (36.5)	76.5 (69.6 to 82.5)**^††^**	0.3 (−8.5 to 9.1)
Not required or recommended	524 (23.4)	46.0 (33.7 to 58.7)**^††^**	611 (19.5)	48.1 (40.3 to 55.9)**^††^**	2.1 (−12.6 to 16.8)
**Receipt of ≥1 dose of a COVID-19 vaccine**					
Yes	1,780 (68.2)	87.6 (83.4 to 91.1)**^††^**	3,361 (89.9)	85.5 (81.8 to 88.7)**^††^**	−2.1 (−7.3 to 3.1)
No (Ref)	609 (31.8)	51.0 (41.7 to 60.2)	257 (10.1)	29.4 (21.2 to 38.8)	−21.6 (−34.4 to −8.8)^¶^

**Abbreviation**: Ref = referent group.

* Respondents were recruited from two preexisting national opt-in Internet sources: Medscape, a medical website managed by WebMD Health Professional Network, and general population Internet panels operated by Dynata.

^†^ Korn-Graubard 95% CI.

^§^ Estimate does not meet CDC’s National Center for Health Statistics standards of reliability (https://www.cdc.gov/nchs/data/series/sr_02/sr02_175.pdf). These estimates are presented in this report for comparison purposes and should be interpreted with caution.

^¶^ Statistically significant (p<0.05) when compared across seasons. The difference between percentages is based on unrounded percentages in each season.

** Race and ethnicity were self-reported. Respondents who identified as Hispanic or Latino might be of any race. The “Other” race category included persons who identified as Asian, American Indian or Alaska Native, Native Hawaiian or other Pacific Islander, and persons who selected “Other” or “multiple races.”

^††^ Statistically significant (p<0.05) when compared with Ref in the same season. The difference between percentages is based on unrounded percentages in each season.

^§§^ Excludes students (34).

^¶¶ ^Includes dentists, allied health professionals, technicians and technologists, emergency technicians, emergency medical technicians, and paramedics.

*** Includes administrative support staff members and managers, and nonclinical support staff members.

^†††^ Respondents could select more than one work setting. Each work setting is represented by a separate variable with two values (yes and no, where reference value is no).

^§§§^ Nursing home, assisted living facility, other long-term care facility, home health agency, or home health care.

^¶¶¶^ Includes dentist office or dental clinic, pharmacy, emergency medical services, and other settings where clinical care or related services were provided to patients.

**** Rurality was defined using zip codes in which >50% of the population resides in a nonmetropolitan county, a rural U.S. Census Bureau tract, or both, according to the Health Resources and Services Administration’s definition of rural population. https://www.hrsa.gov/rural-health/about-us/what-is-rural

^††††^ https://www2.census.gov/geo/pdfs/maps-data/maps/reference/us_regdiv.pdf

Overall, 89.9% of HCP reported having received ≥1 dose of a COVID-19 vaccine, and 87.3% reported having completed the primary COVID-19 vaccination series (Table 2). Among those who completed the primary series, 67.1% reported having received a COVID-19 booster vaccine dose. Completion of primary COVID-19 vaccination was higher among HCP with more than a college degree (97.0%), those with an associate or bachelor’s degree (87.3%), physicians (98.7%), those who received an influenza vaccination during the 2020–21 influenza season (94.1%), and those working in hospitals (91.6%), nonrural areas (88.6%), and facilities where their employer required COVID-19 vaccination (93.1%) compared with the respective reference groups. Similar patterns were observed for receipt of a COVID-19 booster vaccine dose, with the addition of higher coverage among HCP aged 45–59 years (71.7%) and ≥60 years (87.0%), and lower coverage among female HCP (64.7%) and those working in the U.S. Census Bureau South Region (59.8%).

**TABLE 2:** Receipt of ≥1 COVID-19 vaccine dose, completion of primary series,* and receipt of 1 COVID-19 booster dose^†^ among health care personnel, by selected characteristics — Internet panel surveys,^§^ United States, April 2022

Characteristic	Total no. (weighted %) (N = 3,618)	Weighted % (95% CI)^¶^		
		Received ≥1 dose of COVID-19 vaccine (N = 3,618)	Completed primary COVID-19 vaccination series (N = 3,618)	Receipt of first COVID-19 booster dose among HCP who completed primary COVID-19 vaccination series (N= 3,300)
**Overall**	**3,618**	**89.9 (88.2–91.5)**	**87.3 (85.4–89.1)**	**67.1 (63.6–70.4)**
**Age group, yrs**				
18–29 (Ref)	**343 (17.3)**	91.1 (86.4–94.6)	86.4 (80.2–91.3)	50.9 (37.2–64.5)
30–44	**1,616 (39.7)**	88.5 (85.2–91.2)	84.8 (81.3–87.9)	63.1 (58.8–67.3)
45–59	**1,112 (29.1)**	89.7 (86.6–92.2)	88.9 (85.9–91.5)	71.7 (66.8–76.2)**
≥60	**547 (13.9)**	93.2 (88.6–96.4)	92.0 (87.2–95.4)	87.0 (81.8–91.1)**
**Race and ethnicity^††^**				
White, non-Hispanic (Ref)	**2,329 (60.7)**	89.2 (86.8–91.2)	87.1 (84.6–89.3)	66.4 (61.7–71.0)
Black, non-Hispanic	**319 (16.5)**	88.9 (83.6–93.0)	84.9 (78.6–89.9)	60.1 (51.8–68.1)
Hispanic or Latino	**485 (14.3)**	92.1 (87.2–95.6)	88.4 (82.8–92.7)	68.8 (60.2–76.6)
Other, non-Hispanic	**471 (8.5)**	94.4 (89.8–97.4)**	92.1 (85.8–96.2)	81.3 (73.5–87.6)**
**Sex**				
Male (Ref)	**1,081 (21.9)**	92.2 (87.3–95.7)	90.0 (85.0–93.8)	75.2 (69.1–80.7)
Female	**2,537 (78.1)**	89.3 (87.4–91.0)	86.5 (84.4–88.5)	64.7 (60.5–68.6)**
**Education**				
Some college education or less (Ref)	**526 (27.3)**	81.0 (76.3–85.1)	77.5 (72.6–81.9)	50.8 (43.2–58.4)
Associate or bachelor’s degree	**1,038 (45.0)**	90.6 (87.9–92.8)**	87.3 (84.1–90.0)**	65.9 (59.7–71.7)**
Master’s, professional, or doctoral degree	**2,053 (27.7)**	97.7 (96.5–98.6)**	97.0 (95.6–98.0)**	81.5 (77.7–84.9)**
**Occupation^§§^**				
Physician (Ref)	**591 (3.6)**	98.7 (97.3–99.5)	98.7 (97.3–99.5)	89.6 (86.6–92.2)
Nurse practitioner/Physician assistant	**333 (1.7)**	95.1 (92.2–97.2)**	93.8 (90.5–96.1)**	72.8 (67.0–78.1)**
Nurse	**362 (18.7)**	95.4 (92.7–97.3)**	92.8 (88.8–95.7)**	75.5 (69.2–81.1)**
Pharmacist	**509 (1.5)**	96.6 (94.3–98.2)**	96.2 (93.8–97.8)**	83.0 (79.3–86.4)**
Other clinical personnel^¶¶^	**916 (18.8)**	95.2 (92.2–97.3)**	93.9 (90.9–96.2)**	73.4 (69.1–77.5)**
Assistant/Aide	**540 (24.8)**	79.9 (75.9–83.5)**	76.5 (72.4–80.3)**	51.1 (45.3–56.8)**
Nonclinical personnel***	**333 (30.9)**	89.8 (85.2–93.3)**	86.4 (81.4–90.5)**	64.5 (54.6–73.5)**
**Work setting^†††^**				
Hospital	**1,488 (40.3)**	94.1 (91.2–96.3)**	91.6 (88.4–94.2)**	72.7 (68.3–76.7)**
Ambulatory care	**1,335 (31.2)**	92.0 (89.0–94.5)	89.5 (86.1–92.3)	66.4 (61.3–71.2)
Long-term care facility/Home health care^§§§^	**646 (29.3)**	83.6 (79.6–87.0)**	80.0 (75.6–84.0)**	61.0 (51.5–69.9)
Other clinical setting^¶¶¶^	**754 (9.5)**	87.9 (81.4–92.7)	84.8 (77.6–90.3)	63.9 (54.9–72.3)
Location of primary workplace****				
Rural (Ref)	**496 (14.8)**	82.5 (77.1–87.2)	80.6 (75.0–85.4)	62.5 (55.2–69.4)
Nonrural	**3,117 (85.2)**	91.3 (89.5–93.0)**	88.6 (86.5–90.4)**	67.8 (63.8–71.5)
**U.S. Census Bureau region^††††^**				
Northeast (Ref)	**791 (19.9)**	90.4 (86.5–93.4)	89.4 (85.4–92.6)	71.7 (65.3–77.6)
Midwest	**816 (23.1)**	90.7 (86.1–94.2)	87.8 (83.0–91.7)	66.3 (60.3–72.0)
South	**1,251 (35.8)**	88.7 (85.5–91.3)	84.7 (81.0–87.9)	59.8 (52.7–66.6)**
West	**760 (21.1)**	90.8 (86.9–93.9)	89.1 (85.0–92.5)	75.1 (67.7–81.6)
**Employer COVID-19 vaccination recommendation**				
Required (Ref)	**2,155 (59.9)**	95.4 (93.3–97.0)	93.1 (90.8–95.0)	70.4 (67.0–73.8)
Recommended	**1,179 (32.1)**	86.3 (82.6–89.4)**	83.3 (79.2–86.8)**	63.4 (57.1–69.4)**
Not recommended or required	**245 (8.0)**	64.9 (56.2–72.9)**	60.9 (51.9–69.3)**	61.6 (49.6–72.6)
**Receipt of influenza vaccine during 2020–21**				
Yes	**3,115 (79.9)**	96.3 (94.7–97.5)**	94.1 (92.3–95.6)**	71.0 (68.0–73.9)**
No (Ref)	**503 (20.1)**	64.7 (57.0–71.9)	60.3 (52.1–68.1)	42.4 (29.1–56.6)
**Place of first dose of COVID-19 vaccination**				
At work	**1,900 (50.5)**	NA	NA	NA
Place other than work^§§§§^	**1,461 (49.5)**	NA	NA	NA

**Abbreviations:** HCP = health care personnel; NA = not applicable; Ref = referent group.

* Completion of primary series of COVID-19 vaccines was defined as the receipt of a 2-dose primary mRNA COVID-19 vaccine series for respondents who did not report being immunocompromised, or an additional dose after completion of a 2-dose mRNA COVID-19 vaccine series for respondents who reported being immunocompromised. For respondents whose initial vaccine was Janssen (Johnson & Johnson), completion of primary COVID-19 vaccination series was defined as the receipt of 1 dose for those who were not immunocompromised, or a second COVID-19 vaccine (either Janssen or mRNA) for those who were immunocompromised.

^†^ COVID-19 booster vaccination was defined as the receipt of a third dose of COVID-19 vaccine after completion of a 2-dose primary mRNA COVID-19 vaccine series for respondents who did not report being immunocompromised, or a fourth dose of COVID-19 vaccine after completion of a 3-dose mRNA COVID-19 vaccine series for respondents who reported being immunocompromised. For respondents whose initial vaccine was Janssen, booster vaccination was defined as the receipt of a second COVID-19 vaccine dose (either Janssen or mRNA) for respondents who were not immunocompromised or 3 total doses for respondents who were immunocompromised.

^§^ Respondents were recruited from two preexisting national opt-in Internet sources: Medscape, a medical website managed by WebMD Health Professional Network, and general population Internet panels operated by Dynata.

^¶^ Korn-Graubard 95% CI.

** Statistically significant (p<0.05) when compared with Ref.

^††^ Race and ethnicity were self-reported. Respondents who identified as Hispanic or Latino might be of any race. The “Other” race category included persons who identified as Asian, American Indian or Alaska Native, Native Hawaiian or other Pacific Islander, and persons who selected “Other” or “multiple races.”

^§§^ Excludes students (34).

^¶¶^ Includes dentists, allied health professionals, technicians and technologists, emergency technicians, emergency medical technicians, and paramedics.

*** Includes administrative support staff members and managers, and nonclinical support staff members.

^†††^ Respondents could select more than one work setting. Each work setting is represented by a separate variable with two values (yes and no, where reference value is no).

^§§§^ Nursing home, assisted living facility, other long-term care facility, home health agency, or home health care.

^¶¶¶^ Includes dentist office or dental clinic, pharmacy, emergency medical services, and other settings where clinical care or related services were provided to patients.

**** Rurality was defined using zip codes in which >50% of the population resides in a nonmetropolitan county, a rural U.S. Census Bureau tract, or both, according to the Health Resources and Services Administration’s definition of rural population. https://www.hrsa.gov/rural-health/about-us/what-is-rural

**^††††^** https://www2.census.gov/geo/pdfs/maps-data/maps/reference/us_regdiv.pdf

^§§§§^ “Other” place of first or only COVID-19 vaccination includes other medically or nonmedically related place, such as a drugstore, supermarket, and pharmacy.

Employer requirements for receipt of influenza and COVID-19 primary series vaccination were reported by 43.9% and 59.9% of HCP, respectively (Figure). Overall, among HCP who completed the primary series of COVID-19 vaccines, 23.5% reported employer requirement for COVID-19 booster vaccination. HCP working in LTC settings were less likely to report requirements for receipt of any vaccine compared with HCP working in hospitals and ambulatory care settings. Coverage with influenza vaccine, the primary COVID-19 series, and a COVID-19 booster dose was higher among HCP who reported an employer requirement for vaccination than among those who reported an employer recommendation or neither a recommendation nor requirement for vaccination. Among HCP who reported employer vaccination requirements, influenza vaccination coverage ranged from 95.8% to 97.3%, COVID-19 primary series vaccination coverage ranged from 90.2% to 95.1%, and COVID-19 booster vaccination coverage among HCP who completed the primary series of COVID-19 vaccines ranged from 76.4% to 87.8%, by work setting. Among HCP who reported that their employer neither recommended nor required vaccinations, influenza vaccination coverage ranged from 40.1% to 64.2%, COVID-19 primary series vaccination coverage ranged from 54.4% to 62.8%, and among HCP who completed the primary series of COVID-19 vaccines, COVID-19 booster vaccination coverage ranged from 46.1% to 59.7%, by work setting.

**FIGURE:**
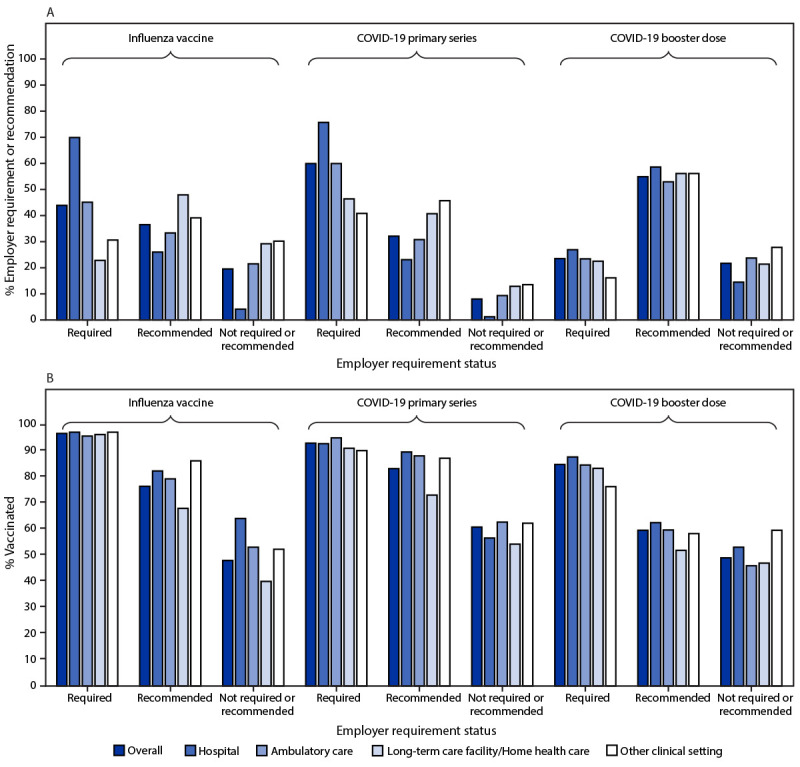
Prevalence of employer requirement or recommendation for influenza and COVID-19* vaccination (A) and vaccination coverage,^†^ by employer requirement status (B) among health care personnel, by work setting^§^ — Internet panel surveys,^¶^ United States, April 2022 * COVID-19 booster vaccination coverage was restricted to health care personnel who completed the primary series of COVID-19 vaccines. Analysis specific to employer requirements for COVID-19 booster vaccines was restricted to 2,256 health care personnel who completed the primary series of COVID-19 vaccines and excluded 1,044 health care providers who encountered an erroneous skip pattern which was corrected on the second day of the survey. ^†^ Completion of primary series of COVID-19 vaccines was defined as the receipt of a 2-dose primary mRNA COVID-19 vaccine series for respondents who did not report being immunocompromised, or an additional dose after completion of a 2-dose mRNA COVID-19 vaccine series for respondents who reported being immunocompromised. For respondents whose initial vaccine was Janssen (Johnson & Johnson), completion of primary COVID-19 vaccination series was defined as the receipt of 1 dose for those who were not immunocompromised, or a second COVID-19 vaccine (either Janssen or mRNA) for those who were immunocompromised. ^§^ Includes dentist office or dental clinic, pharmacy, emergency medical services, and other settings where clinical care or related services were provided to patients. ^¶^ Respondents were recruited from two preexisting national opt-in Internet sources: Medscape, a medical website managed by WebMD Health Professional Network, and general population Internet panels operated by Dynata.

## Discussion

Overall influenza vaccination coverage among HCP during the 2021–22 season was similar to that during the previous season. As observed during previous influenza seasons, nonclinical personnel, assistants and aides, HCP working in LTC settings, HCP with less than a college degree, and HCP who reported their employer neither required nor recommended the influenza vaccine had the lowest vaccination coverage (*5*). Similar patterns were observed for COVID-19 vaccination coverage, although coverage with the primary COVID-19 vaccination series was ≥80% in all work settings, including LTC settings, possibly, in part, because of the prioritization of HCP when the U.S. vaccination program commenced in December 2020 and a relatively high prevalence of employers required COVID-19 vaccination among HCP. Although the prevalence of reported requirements for influenza vaccination during the 2021–22 season increased by approximately 10 percentage points compared with those during the 2020–21 season, requirements for influenza vaccination were lower than were those for COVID-19 vaccination in most work settings, especially LTC settings. Requirements for COVID-19 booster vaccination were infrequently reported in all work settings by HCP who had completed the primary COVID-19 vaccination series, even among hospitals, a large percentage of which had requirements for influenza and COVID-19 primary vaccination. Thus, compared with primary COVID-19 vaccination coverage, influenza vaccination coverage was lower in nonhospital settings, and COVID-19 booster vaccination coverage was lower in all settings. Given that vaccine-induced immunity wanes over time after vaccination, remaining up to date with all COVID-19 recommended vaccination is important for all eligible persons to prevent COVID-19–related hospitalization and severe outcomes, and for HCP to protect their patients (*3*,*8*). In September 2022, CDC recommended an updated bivalent COVID-19 booster vaccination to provide enhanced protection against circulating strains of COVID-19 (*9*).

The findings in this report are subject to at least four limitations. First, the study used a nonprobability sample of volunteer members of Medscape and Dynata Internet panels. Responses were weighted to be representative of the U.S. population of HCP; however, some bias might remain in the coverage estimates. Second, the self-selection of respondents to the panels and to the survey might introduce selection bias if participation in the panel or survey is related to likelihood of being vaccinated. Third, vaccination status was self-reported and might be subject to recall or social desirability bias. Finally, insufficient sample size resulted in the coverage estimates in some subgroups not meeting the National Center for Health Statistics reliability criteria for reporting proportions.

HCP coverage with influenza vaccine, the primary COVID-19 vaccination series, and a booster COVID-19 dose was highest among those who reported employer vaccination requirements for the respective vaccines. Work settings that successfully implemented requirements for primary COVID-19 vaccination could consider the same requirements for COVID-19 booster doses to restore protection among HCP that has declined since their previous vaccination. In addition, many LTC settings now have experience implementing COVID-19 vaccine requirements and could consider these requirements for influenza vaccination to improve influenza vaccination coverage. The Centers for Medicare & Medicaid Services requires that many health care settings report both influenza^§§§^ and COVID-19^¶¶¶^ HCP vaccination data to CDC’s National Healthcare Safety Network; the interim final rule published by the Centers for Medicare & Medicaid Services also requires LTC settings to offer the COVID-19 vaccine to staff members and residents and to educate them about benefits and potential side effects, which might increase vaccination coverage in these settings.**** In addition, useful resources that can help to increase vaccination coverage among HCP include CDC’s long term care web-based toolkit,^††††^ which provides access to resources, strategies, and educational materials, and interventions recommended by the Community Preventive Services Task Force and CDC (*4*,*10*). Annual influenza vaccination and staying up to date with recommended COVID-19 vaccines are critical in prevention of severe disease as well as reduction of influenza and COVID-19–related morbidity and mortality among HCP and their patients.

**Summary**

**What is already known about this topic?**

Influenza and COVID-19 vaccines are recommended for all persons aged ≥6 months, including health care personnel (HCP).

**What is added by this report?**

HCP influenza vaccination coverage was 79.9% during the 2021**–**22 season; 87.3% completed primary COVID-19 vaccination, 67.1% of whom received a COVID-19 booster dose. Influenza, primary COVID-19, and COVID-19 booster coverage was higher among HCP who reported employer vaccination requirements for those vaccines; coverage was lowest among HCP working in long-term care settings.

**What are the implications for public health practice?**

Enhanced efforts are needed to improve HCP vaccination coverage, especially with COVID-19 booster doses and annually for influenza vaccines. Staying up to date with COVID-19 and influenza vaccines can protect HCP and their patients.

## Footnotes

* Physicians, nurse practitioners, physician assistants, nurses, dentists, pharmacists, allied health professionals, technicians, and technologists were recruited from the current membership roster of Medscape. https://www.medscape.com

† Assistants, aides, and nonclinical personnel (e.g., administrators, clerical support workers, janitors, food service workers, and housekeepers) were recruited from general population Internet panels operated by Dynata. https://www.dynata.com

§ Population control totals of U.S. HCP by occupation and work setting were obtained from the U.S. Department of Commerce Bureau of Labor Statistics’ occupational employment and wage statistics (https://www.bls.gov/oes/current/oessrci.htm). Population control totals by other demographic characteristics were obtained from the Bureau of Labor Statistics’ labor force statistics from the current population survey. https://www.bls.gov/cps/data.htm

¶ Major occupational categories included physicians and dentists, nurse practitioners and physician assistants, nurses, pharmacists, other clinical personnel (including allied health professionals, technicians and technologists, and emergency medical technicians and paramedics), assistants and aides, and nonclinical personnel (including administrative support staff members and managers, and nonclinical support staff members).

** A survey response rate requires specification of the denominator at each stage of sampling. During recruitment of an online opt-in survey sample, such as the Internet panels described in this report, these numbers are not available; therefore, a response rate cannot be calculated. Instead, the survey completion rate is provided.

†† 45 C.F.R. part 46; 21 C.F.R. part 56; 42 U.S.C. Sect. 241(d); 5 U.S.C. Sect. 552a; 44 U.S.C. Sect. 3501 et seq.

§§ Completion of primary series of COVID-19 vaccines was defined as the receipt of a 2-dose primary mRNA COVID-19 vaccine series for respondents who did not report being immunocompromised, or an additional dose after completion of a 2-dose mRNA COVID-19 vaccine series for respondents who reported being immunocompromised. For respondents whose initial vaccine was Janssen (Johnson & Johnson), completion of primary COVID-19 vaccination series was defined as the receipt of 1 dose for those who were not immunocompromised, or a second COVID-19 vaccine (either Janssen or mRNA) for those who were immunocompromised (because of solid organ transplant, blood or bone marrow transplant, immune deficiencies, HIV, use of corticosteroids, or use of other immune-weakening medicines).

¶¶ COVID-19 booster vaccination was defined as the receipt of a third dose of COVID-19 vaccine after completion of a 2-dose primary mRNA COVID-19 vaccine series for respondents who did not report being immunocompromised, or a fourth dose of COVID-19 vaccine after completion of a 3-dose mRNA COVID-19 vaccine series for respondents who reported being immunocompromised. For respondents whose initial vaccine was Janssen, booster vaccination was defined as the receipt of a second COVID-19 vaccine (either Janssen or mRNA) for respondents who were not immunocompromised or 3 total doses for respondents who were immunocompromised.

*** Questions included, “Since July 1, 2021, has your employer recommended or required that you be vaccinated for flu?,” “Since December 2020, has your employer recommended or required that you be vaccinated for COVID-19?,” and “Has your employer recommended or required that you be vaccinated with a COVID-19 booster vaccine?” Analyses of employer requirements for COVID-19 vaccine booster doses were restricted to 2,256 HCP who completed the primary series and excluded 1,044 HCP who encountered an erroneous skip pattern, which was corrected on the second day of the survey.

†††
https://www.aapor.org/AAPOR_Main/media/MainSiteFiles/NPS_TF_Report_Final_7_revised_FNL_6_22_13.pdf ; https://www.aapor.org/getattachment/Education-Resources/For-Researchers/AAPOR_Guidance_Nonprob_Precision_042216.pdf

§§§
https://www.cdc.gov/nhsn/pdfs/cms/cms-reporting-requirements.pdf

¶¶¶
https://www.cdc.gov/nhsn/pdfs/covid19/ltcf/cms-covid19-req-508.pdf

****
https://www.cms.gov/medicareprovider-enrollment-and-certificationsurveycertificationgeninfopolicy-and-memos-states-and/interim-final-rule-covid-19-vaccine-immunization-requirements-residents-and-staff

††††
https://www.cdc.gov/flu/toolkit/long-term-care/index.htm
